# Opening up communications

**DOI:** 10.1038/ncomms6523

**Published:** 2014-10-20

## Abstract

The transition to fully open access publishing establishes *Nature Communications* as the flagship *Nature*-branded open access journal.

*Nature Communications* has come a long way since our first submission five years ago. We have now published more than 5,000 papers, 2,000 in 2014 alone. Our editorial team has grown from just 4 to over 30 staff spanning three continents, and our expertise now covers the breadth of the natural sciences. This tremendous expansion, together with our growing recognition within the scientific community, would not have been possible without the trust of our authors and the boundless efforts of our referees and Editorial Advisory Panel, for which we are truly grateful.

We believe in driving open access publishing forwards so that the widest possible audience can appreciate the high quality research we publish.

The journal launched with a ‘hybrid' access model: authors could choose whether content was published for subscribers or as open access through the payment of an article processing charge (APC). This was a bold step for the *Nature* journals—a journal that enabled open access publishing while applying *Nature* standards of editorial evaluation and peer review was without precedent. However, the astonishing growth of open access content at *Nature Communications* suggested that demand was indeed high for a venue where rigorous peer review could be coupled with open access publication.

We are therefore delighted to announce that from today, 20 October 2014, all papers submitted to *Nature Communications* will, if accepted, be published open access through payment of an APC. The default publication licence is Creative Commons Attribution (CC BY). This allows maximum re-use and discovery of a body of work—in the full spirit of open access—although other licence types are available on request.

Naturally, we will honour the preference of authors whose papers have already been submitted, and they will continue to have the choice of subscription or open access options should their work be accepted. In addition, all subscription-access articles will continue to be available to subscribers throughout 2015.

We believe in driving open access publishing forwards so that the widest possible audience can appreciate the high quality research we publish. Independent analysis of our content has shown that by allowing unrestricted access to a paper at the point of publication, open access results in significantly more views and downloads (http://www.nature.com/press_releases/ncomms-report.html). In addition, most major science funders now explicitly allocate funds to pay for open access, but we acknowledge that some authors don't have access to such resources. Consequently, we offer APC waivers for authors from HINARI-eligible countries. We also provide a support service to help authors discover and apply for open access funding from institutions and funders worldwide (http://www.nature.com/authors/open_access/funding.html).

We hope you agree that moving fully open access is a positive step for our authors, readers and the communities we serve. As Nature Publishing Group's flagship open access title, and a premier multidisciplinary journal, we are as committed as ever to delivering a great service to our authors and readers by publishing some of the most interesting scientific discoveries. By making these discoveries accessible to everyone, we hope to further drive the endeavours and advances that they seed.

Published: 20 October 2014

